# Validation and optimization of the Systemic Inflammation-Based modified Glasgow Prognostic Score in predicting postoperative outcome of inflammatory bowel disease: preliminary data

**DOI:** 10.1038/s41598-017-18771-3

**Published:** 2018-01-15

**Authors:** Chenyan Zhao, Chao Ding, Tingbin Xie, Tenghui Zhang, Xujie Dai, Yao Wei, Yi Li, Jianfeng Gong, Weiming Zhu

**Affiliations:** 1 Department of General Surgery, Jinling Hospital, Medical School of Nanjing University, Nanjing, China; 2 Department of General Surgery, Drum Tower Hospital, Medical School of Nanjing University, Nanjing, China

## Abstract

Systemic Inflammation-Based modified Glasgow Prognostic Score (mGPS) was developed as an objective tool to grade state of inflammation. However, the association between mGPS and postoperative complications for inflammatory bowel disease (IBD) patients was still unknown. In our study, 270 IBD patients [Crohn’s disease (CD), n = 186; Ulcerative colitis (UC), n = 84] from January 2013 and January 2016 who underwent elective bowel resection were retrospectively analyzed, and, the levels of preoperative C-reactive protein (CRP) and albumin were included as parameters of mGPS. The incidence of overall postoperative complications was 44.81% (121/270), including 46.77% (87/186) of CD and 40.48% (34/84) of UC. According to multivariate analysis, mGPS (CD: OR = 3.47, p = 0.003; UC: OR = 3.28, p = 0.019) was independently associated with an increased risk of postoperative complications. Patients with a higher mGPS also suffered longer postoperative stay and increased SSIs (both p < 0.05). Combining mGPS with neutrophil ratio improved its prognostic value with a better area under the curve (AUC), using receiver operating characteristic (ROC) method. Then we confirmed that mGPS was associated with postoperative complications in IBD patients undergoing elective bowel resection and the addition of neutrophil ratio enhanced its prognostic value.

## Introduction

Patients suffered from inflammatory bowel disease (IBD) are mainly young adults without significant comorbidities^1–3^, however, the incidence of postoperative complication is higher than other gastrointestinal benign diseases, which may be caused by long-term consumption, inflammatory activity, chronic infection and drug-related adverse effects. Postoperative complications can cause various problems, such as prolonged hospitalization, increased treatment costs, delayed physical function recovery and increased long-term recurrence rate^4–7^. Therefore, early and reliable markers to predict postoperative complications are needed to allow for early identification and intervention.

The risk factors of postoperative complications of IBD are multiform. For Crohn’s disease (CD), low level of preoperative nutritional indicators such as body mass index (BMI), hemoglobin and albumin are independent risk factors of postoperative complications^8–10^. Elevated level of C-reactive protein (CRP) is known to reflect the severity of the disease and contributes to increased risk of postoperative complications, including postoperative intro-abdominal septic complications (IASCs)^11,12^. Infection, abscess or fistula at the time of laparotomy also increases the risk of postoperative complications of CD patients^13^. For ulcerative colitis (UC) patients, anastomotic fistula and pelvic infection are the most common short term complication. Delayed surgery after invalid corticosteroids use, old age (age >60 years), and *Clostridium difficile* infection are the independent risk factors of postoperative complications^14,15^. There is increasing evidence that the presence of a preoperative systemic inflammatory response is a major factor underlying postoperative complications^11,12^. However, a comprehensive and effective system to assess the systemic inflammatory response has not been established for IBD patients.

Modified Glasgow Prognostic Score (mGPS), based on the level of serum CRP and albumin (Table 1), has been effectively used for predicting the outcome of gastrointestinal cancer^16^. Moreover, mGPS has been used in other diseases like systemic lupus erythematosus (SLE) and acute decompensated heart failure (ADHF)^17,18^. It provides more prognostic information in terms of the severity and prognosis for malignant tumor compared to the neutrophil lymphocyte ratio (NLR) and platelet lymphocyte ratio (PLR), which have been widely used in IBD^19–21^.

**Table 1:** Systemic Inflammation-Based Prognostic Scores.

	Points Allocated
Modified GPS (mGPS)	
CRP ≤ 10 mg/L and albumin ≥ 35 g/L	0
CRP >10 mg/L	1
CRP >10 mg/L and albumin <35 g/L	2
GPS = Glasgow Prognostic Score; CRP = C-reactive protein.	

We have two goals for current study. First, we aim to confirm mGPS’s efficacy in predicting short-term postoperative complications in IBD. Second, we aim to optimize the mGPS to enhance its prognostic value for IBD patients.

## Methods

### Patients’ population

This is a single center, retrospective study. Due to the retrospective nature of the study, informed consent was waived, however, this study was performed after approval by the ethics committee of Jinling hospital, and all experiments were performed in accordance with relevant guidelines and regulations.

Information of patients was retrieved from a well maintained IBD database in our center from January 2013 and January 2016. Inclusion criteria were as follows: (1) age 18–65 years, (2) a confirmed diagnosis of IBD according to endoscopy and biopsy, (3) available data on the in-hospital clinical course, (4) patients underwent an elective surgery of IBD-related complications. Exclusion criteria: (1) severe comorbidity and/or organ (kidney, liver or heart) dysfunction, (2) preoperative infections treated with antibiotics, (3) preoperative albumin or blood infusion, (4) surgical procedures for reasons other than IBD-relevant bowel resection.

### Data Collection

Baseline data (BMI, smoking history within 3 months, preoperative medical therapy, Montreal classification, operation details) were collected from the database. Operation details include previous operation history, laparoscopic vs. open surgery, operative time >180 min, estimated blood loss, stoma creation. Laboratory data (1 day before surgery) included CRP, albumin, hemoglobin, platelet, neutrophil ratio. Uncertain or incomplete data were collected by reviewing medical records from the hospital and noted accordingly in the results if not available. According to definition of mGPS (0, 1, and 2), we classified patients into three groups. Patients with both elevated CRP (>10 mg/L) and hypoalbuminemia (<35 g/L) were allocated a score of 2; patients with only CRP >10 mg/L were allocated a score of 1; and patients with neither of these abnormalities were allocated a score of 0 (Table 1).

### Outcomes

Complications were defined as those occurring <30 days after surgery or before hospital discharge, whichever time frame was longer. Based on the Clavien-Dindo system, Grade I-II complications were classified as mild complications, Grade III to IV complications were classified as major complications. An initial complication associated with end-organ failure [acute renal failure, respiratory failure, multiple organ dysfunction syndrome (MODS), unplanned intubation, septic shock, sepsis, cardiac arrest, and death], or unplanned ICU transfer then the initial complication was classified as a grade IV/V complication. Complications not associated with end-organ failure or critical care were considered grade I to III. Surgical site infections (SSIs) included superficial incisional, deep incisional, or organ/space SSIs, such as wound infection, fascia dehiscence, intra-abdominal or pelvic abscess, or anastomotic leakage.

### Statistical Analysis

Statistical analysis was performed with SPSS 20.0 (SPSS, Inc, Chicago, IL). Categorical variables were compared using χ^2^ or Fisher’s exact test. The parametric tests will be applied when normality (and homogeneity of variance) assumptions are satisfied otherwise the equivalent non-parametric test will be used. Parametric variables were analyzed using t-tests, and non-parametric variables were compared using Mann-Whitney U test. A univariate analysis was performed with each potential factor included as an independent variable, and the presence or absence of postoperative complications as the dependent variable. Any variable with a p-value < 0.1 was considered potentially significant and was further analyzed in a stepwise multivariate logistic regression analysis using a backward selection method for determining significant independent factors. The mGPS and mGPS+ Neutrophil ratio were further tested for prognostic value in predicting postoperative complications by logistic regression analysis, and its effectiveness was assessed using area under the receiver operating characteristic curve (AUC). A 2-tailed *p* < 0.05 was considered as statistically significant.

## Results

### Baseline Characteristics

**Figure 1:**
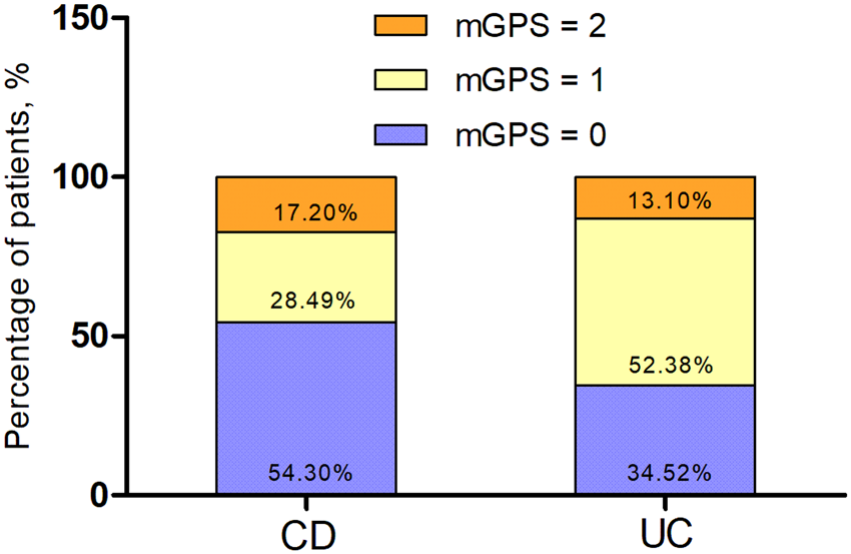
The distribution of IBD patients across the mGPS. CD, Crohn’s disease; UC, Ulcerative colitis; mGPS, modified Glasgow Prognostic Score.

Two hundred and seventy IBD patients were enrolled. Of these, 186 (68.89%) had CD and 84 (31.11%) had UC. In UC, more patients were concentrated in higher mGPS groups (mGPS ≥ 1) as Fig. 1 shows. The prevalence of overall postoperative complications was 44.81% (121/270), including 46.77% (87/186) of CD and 40.48% (34/84) of UC. Overall incidence of mild complications (Clavien-Dindo Grade I–II) was 37.41% (101/270), with 40.86% (76/186) for CD and 29.76% (25/84) for UC. Overall incidence of major complications (Clavien-Dindo Grade III–IV) was 12.59% (34/270), with 11.29% (21/186) for CD and 15.48% (13/84) for UC. Clinical characteristics associated with postoperative complications of the study population were shown in Table 2.

**Table 2:** Univariate Analysis of Baseline Factors associated with Postoperative Complications.

Group	All (n = 270)			CD (n = 186)			UC (n = 84)		
	Non-complications	Complications	P	Non-complications	Complications	P	Non-complications	Complications	P
No. of patients	149	121		99	87		50	34	
Age, years*	34.38 ± 11.41	37.83 ± 13.51	0.024	34.56 ± 10.55	36.13 ± 12.29	0.349	34.04 ± 13.07	42.18 ± 15.58	0.011
Sex, male^†^	76 (51.00%)	81 (66.94%)	0.008	57 (57.58%)	61 (61.62%)	0.076	19 (38.00%)	20 (58.82%)	0.060
BMI (kg/m^2^)*	18.28 ± 2.09	18.30 ± 2.76	0.943	17.68 ± 1.93	17.93 ± 2.29	0.398	19.47 ± 1.88	19.22 ± 3.60	0.681
Disease duration before admission, years*	6.03 ± 6.24	5.63 ± 5.36	0.581	6.11 ± 4.08	5.54 ± 4.13	0.345	5.87 ± 9.18	5.88 ± 7.75	0.998
Smoke within 3 months^†^	3 (2.01%)	8 (6.61%)	0.057	2 (2.02%)	4 (4.60%)	0.321	2 (4.00%)	3 (8.82%)	0.163
mGPS*	0.46 ± 0.64	1.02 ± 0.81	<0.001	0.37 ± 0.58	0.86 ± 0.79	<0.001	0.64 ± 0.72	1.44 ± 0.70	<0.001
CRP >10 mg/L (1 day before surgery)^†^	32 (21.48%)	62 (51.24%)	<0.001	11 (11.11%)	37 (42.53%)	<0.001	21 (42.00%)	25 (73.53%)	0.004
Albumin <35 g/L (1 day before surgery)^†^	38 (25.50%)	60 (49.59%)	<0.001	27 (27.27%)	36 (41.38%)	0.043	11 (22.00%)	24 (70.59%)	<0.001
Neutrophil ratio >75% (1 day before surgery)^†^	13 (8.70%)	33 (27.27%)	<0.001	7 (7.07%)	18 (20.69%)	0.007	6 (12.00%)	15 (44.12%)	0.001
Hemoglobin <12 g/dL (1 day before surgery)^†^	56 (37.58%)	67 (55.37%)	0.004	29 (29.29%)	43 (49.43%)	0.005	27 (54.00%)	24 (70.59%)	0.127
Platelet >400 × 10^9^/L (1 day before surgery)^†^	22 (14.77%)	35 (28.93%)	0.005	10 (10.10%)	15 (17.24%)	0.154	12 (24.00%)	20 (58.82%)	0.001
Preoperative treatment^†^									
5-ASA	5 (3.36%)	10 (8.26%)	0.080	4 (4.04%)	8 (9.20%)	0.153	1 (2.00%)	2 (5.88%)	0.347
Preoperative steroids for ≥ 3 months	6 (4.03%)	30 (24.79%)	<0.001	2 (2.02%)	11 (12.64%)	0.007	4 (8.00%)	19 (55.88%)	<0.001
Immunosuppressant	31 (20.81%)	35 (28.93%)	0.123	25 (25.25%)	29 (33.33%)	0.226	6 (12.00%)	6 (17.65%)	0.468
Infliximab	17 (7.38%)	22 (23.14%)	0.115	5 (5.05%)	9 (10.34%)	0.172	12 (24.00%)	13 (38.24%)	0.161
Enteral nutrition	115 (77.18%)	94 (77.69%)	0.921	89 (100%)	70 (80.46%)	0.068	24 (70.59%)	26 (52.00%)	0.088
Parenteral nutrition	36 (24.16%)	35 (28.93%)	0.376	24 (24.24%)	22 (25.29%)	0.869	12 (24.00%)	13 (38.24%)	0.161
Type of surgery^†^									
Total colectomy	12 (8.05%)	23 (19.01%)	0.008	7 (7.07%)	15 (17.24%)	0.032	5 (10.00%)	8 (23.53%)	0.095
Segmental colectomy	22 (14.77%)	29 (23.97%)	0.055	20 (20.20)	25 (28.74%)	0.175	2 (4.00%)	4 (11.76%)	0.216
IPAA	43 (86.00%)	22 (64.71%)	0.033	—	—	—	43 (86.00%)	22 (64.71%)	0.033
Ileocecal resection	29 (19.46%)	18 (13.95%)	0.323	29 (29.29%)	18 (20.69%)	0.178	—	—	—
Small bowel resection	41 (27.52%)	31 (25.62%)	0.726	41 (41.41%)	31 (35.63%)	0.419	—	—	—
Operation details^†^									
First time operation	105 (70.46%)	78 (64.46%)	0.598	69 (69.70%)	46 (52.87%)	0.018	38 (76.00%)	30 (88.24%)	0.257
Laparoscopic completed cases	76 (51.01%)	49 (40.50%)	0.085	26 (26.26%)	18 (20.69%)	0.372	50 (100.00%)	31 (91.18%)	0.649
Operative time >180 min	83 (55.70%)	64 (52.89%)	0.645	33 (66.67%)	30 (34.48%)	0.869	50 (100.00%)	34 (100.00%)	—
Blood loss, ml	89.97 ± 63.59	93.51 ± 68.59	0.660	101.21 ± 73.86	104.43 ± 74.17	0.768	67.70 ± 23.24	65.59 ± 40.67	0.763
Stoma	78 (52.35%)	52 (42.98%)	0.125	28 (28.28%)	18 (20.69%)	0.231	50 (100.00%)	34 (100.00%)	—

CD = crohn’s disease; UC = ulcerative colitis; BMI = body mass index; mGPS = modified Glasgow Prognostic Score; CRP = C-reactive protein; 5-ASA = 5-aminosalicylic acid; IPAA = ileal pouch-anal anastomosis.

*Values are expressed as the mean ± SD; ^†^values are expressed as n (%).

Patients with postoperative complications tend to be older in UC (42.18 ± 15.58 vs. 34.04 ± 13.07 years, *p* = 0.011), but not in CD (36.13 ± 12.29 vs. 34.56 ± 10.55, *p* = 0.349). As expected, higher mean mGPS among the patients with complications was similarly observed when stratified by CD (0.86 ± 0.79 vs. 0.37 ± 0.58, *p* < 0.001) and UC (1.44 ± 0.70 vs. 0.64 ± 0.72, *p* < 0.001).

Otherwise, patients with or without postoperative complications were similar with regard to sex ratio (CD, *p* = 0.076; UC, *p* = 0.060), BMI (CD, *p* = 0.398; UC, *p* = 0.681), disease duration before admission (CD, *p* = 0.345; UC, *p* = 0.998), smoke habits within 3 months (CD, *p* = 0.321; UC, *p* = 0.163).

### Uni- and Multi-variate Analyses of Factors for Postoperative Complications

In CD, factors found to be significantly associated with postoperative complications in univariate analysis included CRP level >10 mg/l (within 1 day before surgery), albumin level <35 g/L (1 day before surgery), neutrophil ratio >75% (1 day before surgery), hemoglobin <12 g/dL (1 day before surgery), preoperative steroid use for ≥3 months, total colectomy, and first time operation. In UC, the risk factors were age, CRP level >10 mg/l (1 day before surgery), albumin level <35 g/l (1 day before surgery), neutrophil ratio >75% (1 day before surgery), platelet >400 × 10^9^/L (1 day before surgery), preoperative steroid use for ≥3 months, albumin level <35 g/l (1 day before surgery). Factors with *p* < 0.100 were included in multivariate analysis model to determine the risk factors independently associated with the development of postoperative complications.

In multivariate analysis, only mGPS (0/1/2), neutrophil ratio >75% (1 day before surgery), preoperative steroids for ≥3 months was found to be an independent risk factor for postoperative complications both in CD and UC, as shown in Table 3.

**Table 3:** Multivariate Analyses of Factors associated with Postoperative Complications.

	CD				UC			
	Univariate	Multivariate			Univariate	Multivariate		
	p	OR	95% CI	p	p	OR	95% CI	p
Age, years	0.349				0.011	1.103	0.905–1.137	0.994
Sex, male	0.076	1.261	1.024–2.533	0.224	0.060	1.376	0.989–1.719	0.161
mGPS (0/1/2)	<0.001	3.473	1.107–5.330	0.003	<0.001	3.275	1.639–3.784	0.019
Neutrophil ratio >75% (1 day before surgery)	0.007	1.922	1.058–2.491	0.027	0.001	2.265	1.788–2.794	0.031
Hemoglobin <12 g/dL (1 day before surgery)	0.005	1.354	0.756–1.945	0.126	0.127	—	—	—
Platelet >400 × 10^9^/L (1 day before surgery)	0.154	—	—	—	0.001	1.453	0.700–3.559	0.131
Preoperative steroids for ≥ 3 months	0.007	1.843	1.007–1.906	0.045	<0.001	2.166	1.106–3.569	0.036
Enteral nutrition	0.068	0.806	0.563–1.164	0.217	0.088	0.837	0.605–1.294	0.382
Total colectomy	0.032	2.455	1.399–4.013	0.173	0.023	1.963	1.562–3.223	0.096
First time operation	0.018	0.968	0.704–1.996	0.806	0.257	—	—	—

CD = crohn’s disease; UC = ulcerative colitis; OR = odds ratio; mGPS = modified Glasgow Prognostic Score.

### Postoperative complications associated with mGPS

CD patients (n = 186) were allocated as shown in Table 4: 101 (54.30%) to mGPS 0, 53 (28.49%) to mGPS 1, and 32 (17.20%) to mGPS 2. The incidences of overall postoperative complications were 33.66%, 58.49%, and 68.75% for the GPS 0, 1, and 2 groups (*p* < 0.001), respectively. Incidences of mild postoperative complications (*p* = 0.006), major complications (*p* = 0.001) and surgical site infection (p = 0.006) were all significantly different among three groups. The length of postoperative hospital stay elongated as the mGPS increased (17.19 ± 4.15 vs. 18.59 ± 4.25 vs. 20.26 ± 3.49, *p* = 0.001).

**Table 4:** Correlation between postoperative complications and mGPS.

	CD (n = 186)				UC (n = 84)			
	mGPS = 0	mGPS = 1	mGPS = 2	P	mGPS = 0	mGPS = 1	mGPS = 2	P
No. of patients*	101 (54.30%)	53 (28.49%)	32 (17.20%)		29 (34.52%)	44 (52.38%)	11 (13.10%)	
All complications*	34 (33.66%)	31 (58.49%)	22 (68.75%)	<0.001	8 (27.59%)	18 (40.91%)	8 (72.73%)	0.034
Mild complications (Grade I–II)*^‡^	31 (30.69%)	26 (49.06%)	19 (59.36%)	0.006	7 (24.14%)	12 (27.27%)	6 (54.55%)	0.149
Major complications (Grade III or greater)*^‡^	4 (3.96%)	9 (16.98%)	8 (25.00%)	0.001	1 (3.45%)	7 (15.91%)	5 (45.45%)	0.007
Grade III*^‡^	4 (3.96%)	7 (13.21%)	9 (28.13%)		1 (3.45%)	6 (13.64%)	3 (27.27%)	
Abdominal-pelvic abscess	2 (1.98%)	3 (5.66%)	3 (9.38%)		1 (3.45%)	2 (4.55%)	2 (18.18%)	
Pleural effusion	0	1 (1.89%)	2 (6.25%)		0	1 (2.27%)	0	
Anastomotic leakage	1 (0.99%)	1 (1.89%)	3 (9.38%)		0	1 (2.27%)	0	
Gastrointestinal bleeding	0	0	1 (3.13%)		0	2 (4.55%)	1 (9.09%)	
Intra-abdominal bleeding	0	1 (1.89%)	1 (3.13%)		0	0	0	
Stoma complications	1 (0.99%)	1 (1.89%)	1 (3.13%)		0	0	0	
Grade IV*^‡^	0	2 (3.77%)	3 (9.38%)		0	1 (2.27%)	2 (18.18%)	
Septic shock	0	0	1 (3.13%)		0	0	0	
Sepsis	0	1 (1.89%)	0		0	0	0	
Respiratory failure	0	0	1 (3.13%)		0	1 (2.27%)	0	
Kidney failure	0	1 (1.89%)	1 (3.13%)		0	0	1 (9.09%)	
MODS	0	0	0		0	0	1 (9.09%)	
Grade V*^‡^	0	0	1 (3.13%)	0.170	0	0	1 (9.09%)	0.115
SSI (+)*	8 (7.92%)	12 (22.64%)	13 (40.63%)	0.006	1 (3.45%)	6 (13.64%)	4 (36.36%)	0.003
Hospital stay (days)^†^	17.19 ± 4.15	18.59 ± 4.25	20.26 ± 3.49	0.001	21.81 ± 3.66	24.98 ± 3.41	26.83 ± 4.78	0.001

CD = crohn’s disease; UC = ulcerative colitis; MODS = multiple organ dysfunction syndrome; SSI = surgical site infection.

*Values are expressed as the mean ± SD; ^†^values are expressed as n (%); ^‡^Clavien-Dindo’s classification of surgical complication.

UC patients (n = 186) were allocated as follows: 29 (34.52%) to mGPS 0, 44 (52.38%) to mGPS 1, and 11 (13.10%) to mGPS 2. Incidences of overall postoperative complications were 33.66%, 58.49%, and 68.75% for the mGPS 0, 1, and 2 groups (p < 0.001), respectively. Significant different incidences of major complications (*p* = 0.007), surgical site infection (*p* = 0.003) were observed, but not of mild postoperative complications (*p* = 0.149). A prolonged hospital stay was also observed as mGPS increased (21.81 ± 3.66 vs. 24.98 ± 3.41 vs. 26.83 ± 4.78, *p* = 0.001).

### The predictive value of mGPS and its optimization

**Figure 2:**
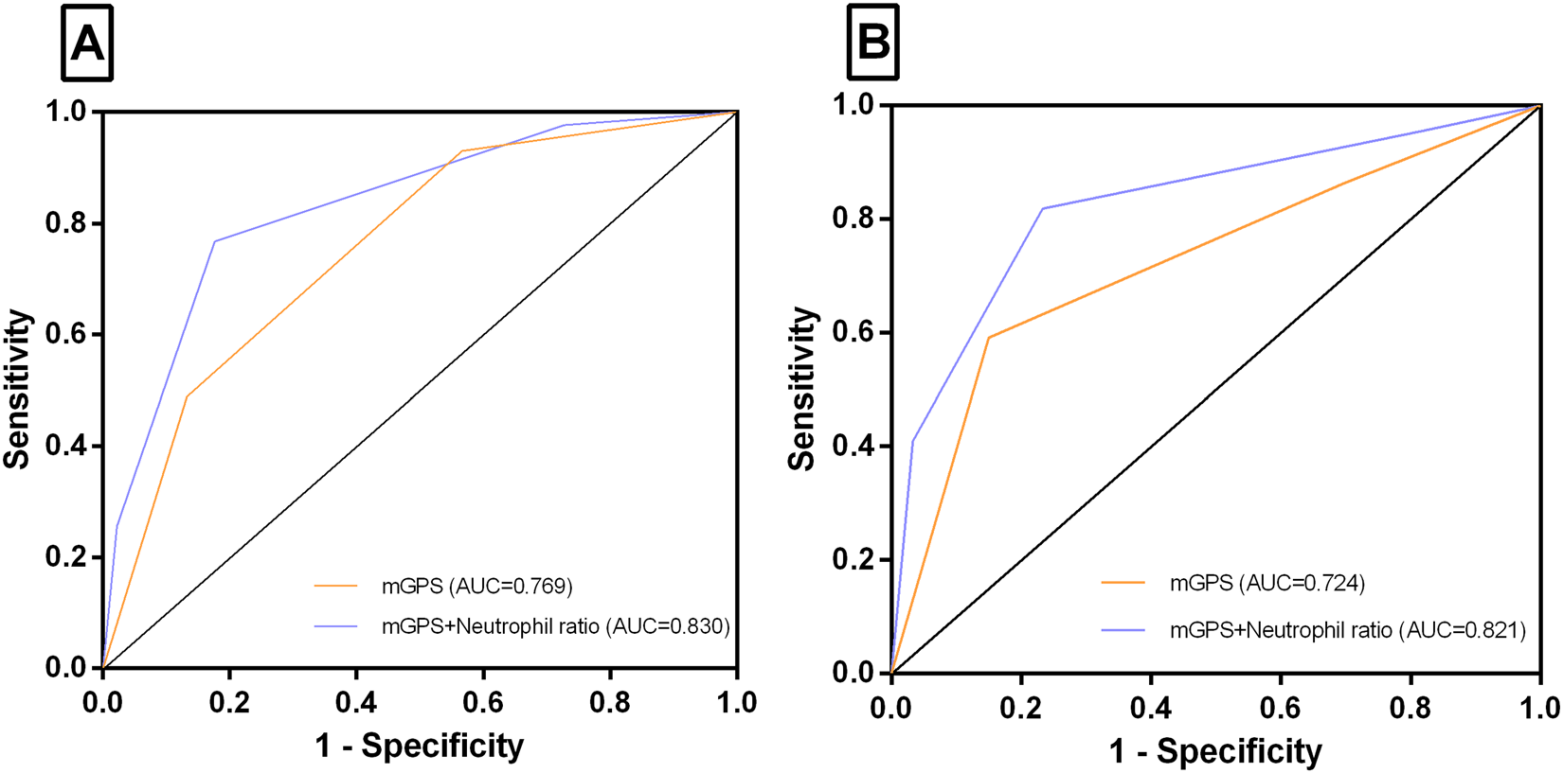
Receiver operating characteristic curves (ROC) for mGPS and mGPS + neutrophil ratio >75% in the prediction of postoperative complications. (**A**) For CD patients, the area under the receiver operating characteristic curve (AUC) for mGPS was 0.769, and AUC for mGPS + neutrophil ratio >75% was 0.830. (**B**) For UC patients, the AUC for mGPS was 0.724, and AUC for mGPS + neutrophil ratio >75% was 0.821.

The odds ratio (OR) and AUC of mGPS in IBD patients for prediction of overall postoperative complications were shown in Table 5 and Fig. 2. Higher mGPS was associated with increased occurrence of postoperative complications. In CD patients, the ORs were 2.72 (95% CI, 1.35–4.89, *p* < 0.001) and 3.94 (95% CI, 2.08–6.91, *p* < 0.001) respectively for mGPS 1 and 2. The AUC was 0.769 (*p* < 0.001). In UC patients, the ORs were 1.48 (95% CI, 1.16–3.34, *p* < 0.001) and 3.64 (95% CI, 1.85–5.77, *p* < 0.001) respectively for mGPS 1 and 2, and AUC was 0.724 (*p* < 0.001).

**Table 5:** Validation and optimization of the mGPS in predicting postoperative complications.

		CD (n = 186)				UC (n = 84)			
		No. (%)	OR (95% CI)	P	AUC	No. (%)	OR (95% CI)	P	AUC
mGPS	0	101 (54.30%)	1 (reference)	<0.001	0.769	29 (34.52%)	1 (reference)	<0.001	0.724
	1	53 (28.49%)	2.72 (1.35–4.89)			29 (34.52%)	1.48 (1.16–3.34)		
	2	32 (17.20%)	3.94 (2.08–6.91)			26 (30.95%)	3.64 (1.85–5.77)		
mGPS	0	91 (48.92%)	1 (reference)	<0.001	0.830	28 (33.33%)	1 (reference)	<0.001	0.821
+Neutrophil ratio >75%	1	60 (32.26%)	2.30 (1.11–3.86)			27 (32.14%)	1.51 (1.09–2.73)		
	2	25 (13.44%)	4.25 (1.96–7.54)			12 (14.29%)	3.99 (1.53–5.47)		
	3	10 (5.38%)	11.86 (6.48–17.49)			17 (20.24%)	9.47 (5.24–15.33)		

CD = crohn’s disease; UC = ulcerative colitis; OR = odds ratio; AUC = area under the curve; mGPS = modified Glasgow Prognostic Score.

Neutrophil ratio >75% was found to be independently associated with the occurrence of postoperative complications in CD (OR: 1.922; 95% CI: 1.058–2.491; *p* = 0.027) and UC (OR: 2.265; 95% CI: 1.788–2.794; *p* = 0.031), then the biomarkers mGPS and neutrophil ratio >75% were assessed as a combined bioscore using a logistic regression model to evaluate the prognostic capacity in predicting the occurrence of postoperative complications. In CD patients, the ORs were 2.30 (95% CI: 1.11–3.86), 4.25 (95% CI: 1.96–7.54) and 11.86 (95% CI: 6.48–17.49) for a bioscore 1, 2 and 3 respectively (p < 0.001). In UC patients, the ORs were 1.51 (95% CI: 1.09–2.73), 3.99 (95% CI: 1.53–5.47) and 9.47 (95% CI: 5.24–15.33) for a bioscore 1, 2 and 3 respectively (p < 0.001). Combination of mGPS and neutrophil ratio >75% was found to improve its prognostic value with a better AUC (all *p* < 0.001).

## Discussion

This study validated and optimized the temporal association between preoperative mGPS and postoperative complications in IBD patients. In the current study, mGPS could act as a tool to offer early identification of critical postoperative complications. Patients with higher mGPS within 1 day before surgery were also at higher risk of prolonged postoperative hospital stay and more SSIs. Combination of mGPS and neutrophil ratio >75% enhanced the prognostic value in the form of a better AUC.

The underlying mechanisms of relationship between an elevated preoperative mGPS and postoperative complications in IBD patients might be explained in several ways. First, the increased CRP levels observed in IBD patients may be due to the increased production of pro-inflammatory cytokines. Indeed, pro-inflammatory factor and immune-regulatory factors like TNF-α and IL-6, which promoting inflammation and playing a role in signal transduction, increased significantly in IBD patients^22^. On the other hand, hypoalbuminemia was associated with impairment of the innate immune response; hypoalbuminemia has been proved to cause impairment of macrophage activation and induce macrophage apoptosis^23,24^, suggesting that the body’s immune defenses were some extent disabled. Also, hypoalbuminaemia reflected loss of lean tissue, which further compromised immune function^25,26^. Therefore, the presence of a systemic inflammatory response, as indicated by elevated CRP level and hypoalbuminemia, should be routinely evaluated prior to surgery.

Our conclusion is well-supported by evidence from previous findings besides our own. As a marker of systemic inflammation before surgery, mGPS was associated with postoperative infection in patients undergoing resection of gastrointestinal cancer with a risk ratio (RR) of 1.89^27^, and was also related to blood transfusion requirements and post-operative complications in hepatic resection for hepatocellular carcinoma^28^. Preoperative CRP >10 mg/l or changes of CRP (ΔCRP) were also risk factors for postoperative IASCs of CD^11^. Albumin, another component of mGPS, combined with BMI and hemoglobin, were important indexes of evaluating the nutritional status of IBD patients^8–10^. Preoperative low albumin significantly increased the risk of septic complications after surgery in CD. In the present study, the rate of overall postoperative complications increased significantly along with the increase of mGPS both for CD and UC patients.

Previous studies have shown that mGPS could provide short-term and long-term prognostic information for various tumors such as lung, breast, esophagus or stomach, pancreas, kidney and colon-rectum carcinoma^29–33^. In the present study, totally 121 (44.81%) patients developed postoperative complications, 87/186 (44.77%) in CD and 34/84 (40.48%) in UC. mGPS was identified as an independent risk factors associated with complications of IBD, the rate of overall postoperative complications increases significantly along with the increase of mGPS. For CD, significant different incidences of mild postoperative complications (p = 0.006), major complications (*p* = 0.001), surgical site infection (*p* = 0.006) and hospital stays (*p* = 0.001) were observed between mGPS groups (mGPS = 0 vs. mGPS = 1 vs. mGPS = 2). For UC, the incidences of major complications (*p* = 0.007), surgical site infection (p = 0.003) and hospital stays (p = 0.001) were also significant different between mGPS groups (mGPS = 0 vs. mGPS = 1 vs. mGPS = 2), except the mild postoperative complications (*p* = 0.149). We speculate that this is because in UC, more patients were concentrated in higher mGPS groups (mGPS ≥ 1) as Fig. 1 shows, then the value of the mGPS to mild postoperative complications likely would be some extent diluted.

Furthermore, our findings suggest that after combined mGPS with an elevated neutrophil ratio (>75%), the overall AUC of mGPS was improved for IBD patients, also, the addition to the mGPS has led to an increase in the ORs associated with overall postoperative complications. Then a refinement of the mGPS with other components of the systemic inflammatory response appeared to improve its prognostic value. This is consistent with recent publications in IBD cohorts. Chikao *et al*. recently reported that circulating neutrophil elastase levels in the early postoperative period might be a useful predictor of postoperative infectious complications in immune-controlled UC patients who received high doses of steroids^34^. Chikao *et al*. also confirmed that preoperative neutrophil activation may be one risk factor for postoperative morbidity when the patients undergo intense surgical stress^35^.

This study had limitations as it was performed retrospectively and from a single center, effects of residual confounding factors could not be fully excluded and perioperative management strategies were dependent on our local experience, which may influence the outcome. Second, we did not explore the relationship between mGPS and long-term prognosis of IBD patients, such as late relapses, readmission and some postoperative long-term complications.

## Conclusion

The current study confirmed mGPS predicted postoperative outcomes in patients undergoing IBD related bowel resection. The addition of neutrophil ratio and hemoglobin in CD; and neutrophil ratio and platelet counts in UC respectively improved the prognostic value and clinical usefulness of the mGPS in IBD patients.
